# Prenatal transcript levels and metabolomics analyses reveal metabolic changes associated with intrauterine growth restriction and sex

**DOI:** 10.1098/rsob.220151

**Published:** 2022-09-14

**Authors:** Siriluck Ponsuksili, Eduard Murani, Frieder Hadlich, Muhammad Arsalan Iqbal, Beate Fuchs, Christina E. Galuska, Alvaro Perdomo-Sabogal, Fabio Sarais, Nares Trakooljul, Henry Reyer, Michael Oster, Klaus Wimmers

**Affiliations:** ^1^ Research Institute for Farm Animal Biology (FBN), Institute for Genome Biology, Wilhelm-Stahl-Allee 2, 18196 Dummerstorf, Germany; ^2^ Research Institute for Farm Animal Biology (FBN), Core Facility Metabolomics, 18196 Dummerstorf, Germany; ^3^ Faculty of Agricultural and Environmental Sciences, University Rostock, 18059 Rostock, Germany

**Keywords:** IUGR, miRNA, transcriptome, metabolome, fetal weight, pig

## Abstract

The metabolic changes associated with intrauterine growth restriction (IUGR) particularly affect the liver, which is a central metabolic organ and contributes significantly to the provision of energy and specific nutrients and metabolites. Therefore, our aim was to decipher and elucidate the molecular pathways of developmental processes mediated by miRNAs and mRNAs, as well as the metabolome in fetal liver tissue in IUGR compared to appropriate for gestational age groups (AGA). Discordant siblings representing the extremes in fetal weight at day 63 post conception (dpc) were selected from F2 fetuses of a cross of German Landrace and Pietrain. Most of the changes in the liver of IUGR at midgestation involved various lipid metabolic pathways, both on transcript and metabolite levels, especially in the category of sphingolipids and phospholipids. Differentially expressed miRNAs, such as miR-34a, and their differentially expressed mRNA targets were identified. Sex-specific phenomena were observed at both the transcript and metabolite levels, particularly in male. This suggests that sex-specific adaptations in the metabolic system occur in the liver during midgestation (63 dpc). Our multi-omics network analysis reveals interactions and changes in the metabolic system associated with IUGR and identified an important biosignature that differs between IUGR and AGA piglets.

## Graphical abstract

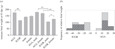

## 1. Introduction

Approximately 15–20% of piglets in each litter are affected by growth restriction in the uterine horn, resulting in low piglet birth weight [1]. Intrauterine growth restriction (IUGR) is a process caused by an inadequate supply of nutrients and oxygen to the fetus due to maternal malnutrition or placental insufficiency resulting in a fetal weight that is two or more standard deviations lower than the mean at the corresponding gestational age [1,2]. All these processes directly affect metabolic organs, including muscle and liver. Our previous study uncovered the molecular pathways involved in skeletal muscle growth and development and their role in IUGR fetus and in fetal weight [3,4]. In addition, transcriptomic analysis in *longissimus dorsi* muscle (LDM) from pig fetuses at 63 days post conception (dpc) revealed miRNAs and their target genes that correlate with fetal weight [5]. Metabolic dysfunction in skeletal muscle and liver of IUGR fetuses, including mitochondrial function, was reported [6]. The impact of IUGR also affects the proteomes of the small intestine, intermediate metabolism in the liver and energy production in skeletal muscle of fetuses and newborns [7,8]. Higher activity of glutamate oxaloacetate transaminase and lower activity of lipoprotein lipase were reported in the liver of IUGR fetus than the normal fetuses [7]. Insights into shifts of the lipid metabolism in both normal-grown and IUGR fetuses are still limited particularly in pig. Hepatic lipid content in IUGR fetal sheep is similar to controls [9], whereas some studies demonstrate increased hepatic lipid accumulation in preterm human neonates [10]. Fetal sheep exhibiting IUGR showed activation of hepatic glucose production (HGP), increased hepatic gluconeogenic gene expression and resistance to the normal suppression of HGP by insulin [9,11]. Moreover, the use of a sheep model of maternal malnutrition revealed a disturbance in fetal liver lipid metabolism and impact on oxidative stress [12,13]. Compared with other metabolic processes, lipid metabolism remains relatively unexplored in both normally developing fetuses and fetuses with IUGR. All fetuses are capable of synthesizing lipids, but the large differences in neonatal lipid content between species suggest that placental transfer of fatty acids and/or their rate of synthesis by fetuses differs. Fatty acid uptake is low in pigs with an epitheliochorial placenta compared with primates and rodents with hemochorial placentas [14]. In addition, fetal pigs have limited synthesis of fatty acids and triacylglycerols (TAG), resulting in a body fat content of only 1% at birth [15,16]. In pigs, previous studies have shown that the majority of fatty acids in fetuses are not directly derived from maternal free fatty acids (FFA). The increase in plasma-free fatty acids in pregnant sows due to fasting did not affect the percentage of body fat in the fetus [17].

The role of miRNAs in the pathophysiology of pregnancy-associated disorders and potential miRNAs biomarkers for pregnancy complications were reported in our recent studies [3,5,18]. We found fetal weight correlated abundances of muscular miRNAs with potential involvement in IUGR [5]. MiR-34a and miR-210 are the most commonly reported miRNAs involved in the pathophysiology of IUGR, targeting genes for muscle growth and fetal development [3,19,20]. MicroRNAs including miR-29a were shown to trigger the impairment of intestinal epithelial integrity during IUGR in pig [21]. Furthermore, let-7 miRNA family members were shown to be closely related to metabolic processes and serum glucose and insulin content [22,23].

However, liver metabolic and transcriptomic adaptations in IUGR are expected to differ from those in muscle tissues. The liver represents the central metabolic organ and contributes significantly to the supply of energy and specific nutrients, metabolites and also bioactive molecules to the peripheral organs. In pigs, a critical time point for myogenesis was determined at about 63 dpc, where the formation of primary myotubes and secondary fibres overlapped [24]. As for the liver, ultrastructural studies revealed that the fetal pig liver exhibits specific hepatic metabolic competence and hepatopoietic activity at 40–80 dpc, followed by strong glycogen accumulation [25]. Therefore, this time point was chosen for muscle in our previous study [3,4] and for liver in this study.

In pigs, a polytocus animal distinct from sheep and humans, litter size and offspring weight act antagonistically. IUGR occurs in a discrete subset of fetuses that are substantially smaller than their littermates. These universal IUGR effects occur in modern pig breeds where selection for larger litters applies to sows from commercial dam lines. In our study, sibling pairs of IUGR or AGA fetuses of the same sex from the same sow were selected. Applications of metabolomics for the study of animal physiology and biochemistry, especially in IUGR, are still limited. Therefore, we aim to use metabolomics and mRNA and miRNA expression analyses to obtain a holistic view of metabolic changes during midgestation in fetuses with IUGR. The analysis of the metabolome and mRNA and miRNA transcript profiles from liver samples of fetuses with 63 dpc, representing growth-restricted and adequately developed phenotypes, aims to identify liver molecular features associated with intrauterine development. Moreover, the mRNA and miRNA expression data and metabolome data were integrated to infer regulatory networks in the liver and to elucidate their contribution to IUGR.

## 2. Results

**Figure 1.**
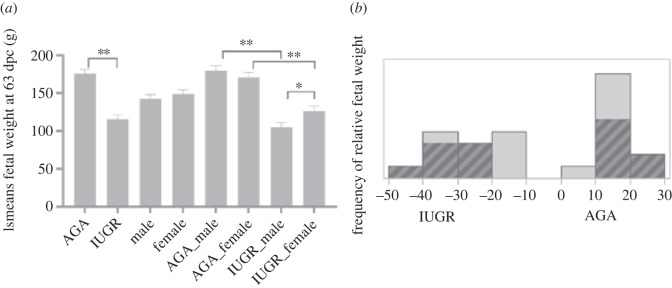
(*a*) Fetal weight at 63 days post conception (dpc) comparing between groups (AGA versus IUGR), sex (male versus female) and the interaction between groups and sex. Fetal weight value (lsmean ± s.e.) are given in grams (g). **p < 0.0001 and *p < 0.001. (*b*) Histogram of relative fetal weight. The dark shadows indicate the proportion of males in this range of relative fetal weight.

The mean weight of the IUGR fetuses was significantly lower than that of the AGA fetuses (115.77 ± 5.5 g versus 175.64 ± 5.5 g; *p* < 0.0001) (figure 1*a*). A significant difference was also found when comparing fetal weight between AGA and IUGR groups in both males and females. No significant difference in weight was found between the sexes across all fetuses, nor between the sexes within the AGA group. By contrast, a weight difference was found between the sexes in the IUGR fetuses (*p* < 0.008), with the mean weight of the male fetuses being lower than that of the females. The histogram of relative fetal weight (%) used to explain the risk of piglet death is shown in figure 1*b*.

### 2.1. Differential expression of mRNA and pathways analysis

A total of 10 086 probe sets passed quality filtering and were used for further analyses. Mixed-model analysis of expression levels between IUGR and AGA groups, sexes and interaction of the groups and sex in the model revealed 1040 probe sets, showing significant differences in at least one of the comparison groups (FDR < 0.1).

**Figure 2.**
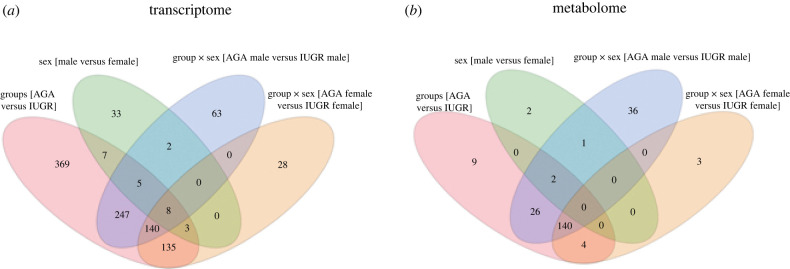
Venn diagrams showing the number of (*a*) transcripts and (*b*) metabolites associated with fetal groups, sex and the interaction of group and sex.

We found that 282 of 914 transcripts were upregulated in IUGR, whereas 632 were upregulated in AGA groups. Few transcripts differed between sexes, including 17 that were upregulated in females and 41 that were upregulated in males. The interaction between sex and fetal outcome revealed interesting aspects. In males, 465 transcripts changed between the AGA and IUGR groups, whereas in females, 314 transcripts changed between the AGA and IUGR groups. Full details of these data can be found in electronic supplementary material, table 1. The number of differentially abundant transcripts between groups and their overlap were shown in figure 2*a*.

**Figure 3.**
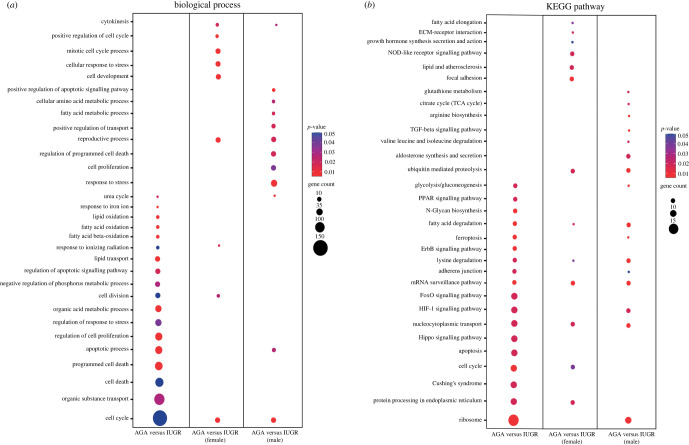
(*a*) Gene Ontology and (*b*) KEGG pathway enrichment analysis of differentially expressed mRNAs between IUGR and AGA and their interaction within sex. The dot size represents the number of transcripts involved in each biological process, while the colour of dots indicates the *p*-value.

The differentially expressed genes from each comparison were subjected to DAVID (version.6.8) for Gene Ontology (biological processes) (figure 3*a*) and KEGG pathway (figure 3*b*) enrichment analysis. Liver transcripts differentially expressed between IUGR and AGA were enriched in biological processes such as cell cycle, cell death, apoptotic processes and lipid metabolic processes. When comparing IUGR and AGA in females, the differentially expressed genes were enriched in cellular processes, whereas in males they were more enriched in metabolic processes.

**Figure 4.**
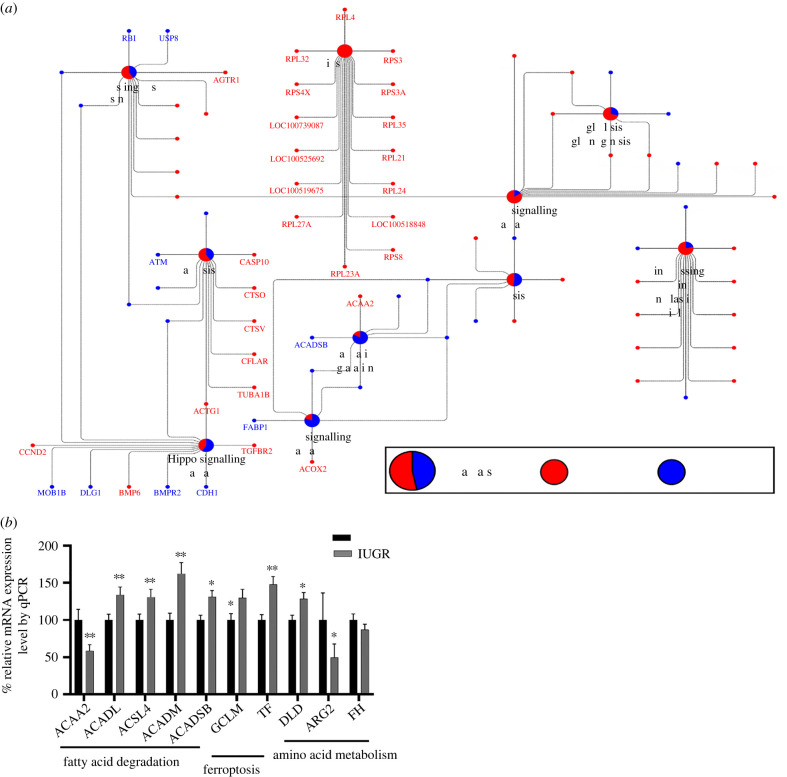
(*a*) KEGG pathway enrichment analysis of differentially expressed mRNAs between IUGR and AGA. The pie charts indicate the group-specific proportions of mRNAs associated with IUGR to the KEGG pathways. The red sections show mRNAs upregulated in IUGR group, whereas the blue sections represent mRNAs downregulated in the IUGR group. (*b*) Expression of genes related to fatty acid degradation, ferroptosis and enzymes of other amino acid metabolism by real-time qPCR.

In KEGG pathways, the genes differentially expressed between IUGR and AGA were enriched in metabolic processes such as glycolysis/gluconeogenesis, PPAR signalling pathway, fatty acid degradation, ribosome, ferroptosis and lysine degradation. Other pathways such as the HIF-1 signalling pathway and the Hippo signalling pathway were also prominent when comparing between IUGR and AGA, particularly in males. Metabolic pathways of interest such as glycolysis/gluconeogenesis, citrate cycle (TCA cycle), ribosome and ferroptosis were more enriched in males than in females when comparing IUGR and AGA. Figure 4*a* shows some selected KEGG pathways. Upregulated transcripts in the IUGR group were enriched in ribosome, HIF-1 signalling pathway, protein processing, glycolysis/gluconeogenesis, apoptosis and Cushing's syndrome. By contrast, the downregulated transcripts in the IUGR groups were enriched in Hippo signalling, PPAR signalling and fatty acid degradation. We selected some of the transcripts of these pathways for validation by qPCR (figure 4*b*).The microarray and qPCR data show high level of correspondence with Pearson correlation coefficient (*r*) of: *ACAA2* (*r* = 0.94, *p* < 0.0001), *ACADL* (*r* = 0.85, *p* < 0.0001, *ACADM* (*r* = 0.70, *p* = 0.0001), *ACADSB* (*r* = 0.55, *p* = 0.0052), *ACSL4* (*r* = 0.69, *p* = 0.0002), *ARG2* (*r* = 0.75, *p* < 0.0001), *DLD* (*r* = 0.62, *p* = 0.0011), *FH* (*r* = 0.58, *p* = 0.0027), *GCLM* (*r* = 0.75, *p* < 0.0001) and *TF* (*r* = 0.65, *p* = 0.0006).

### 2.2. Differential expression of miRNA and their mRNA targets

**Figure 5.**
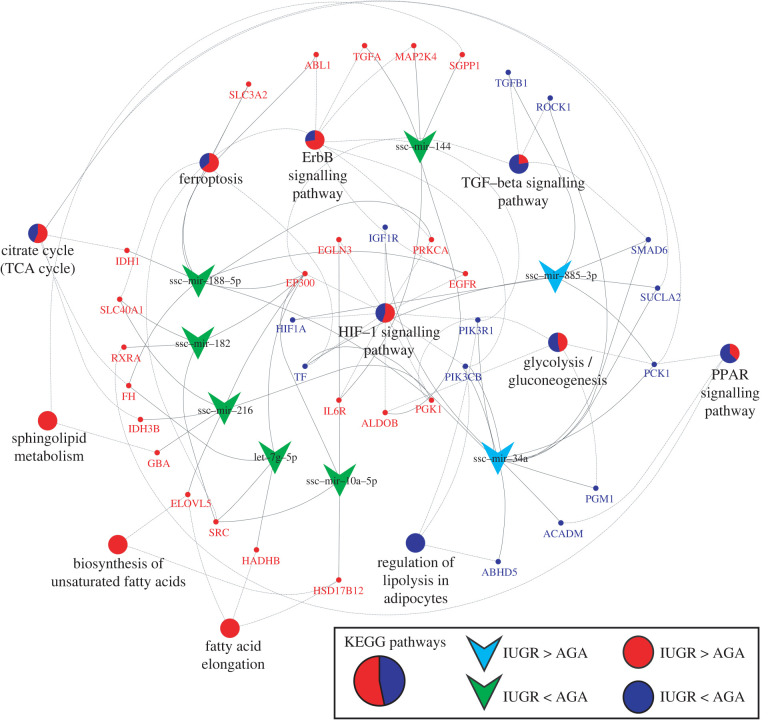
Correlation network analysis of eight significantly regulated miRNAs between IUGR and AGA fetal groups and their negative correlation and predicted mRNA targets. These targets mRNA belong to KEGG pathway. The pie charts indicate the proportion of the gene's contribution to KEGG pathways. The red section shows that mRNAs were upregulated in IUGR compared to AGA, while the blue section showed the opposite. The green symbol depicts miRNAs downregulated in IUGR, whereas the sky blue symbol indicates miRNAs upregulated in IUGR groups.

The miRNAs were selected based on our previous study on fetal muscle samples involving fetuses [3]. A total of 40 miRNAs were used for liver miRNA profiling. Two miRNA (miR-885-3p and miR-34a) were upregulated in the IUGR group compared with the AGA group, while six miRNA (miR-216, miR-188-5p, miR-144, miR-10a-5p, let-7 g-5p and miR-182) were downregulated in the IUGR groups. Only miR-155-5p was significantly upregulated in males compared with females. The miRNAs ssc-miR-216, miR-188-5p, miR-885-3p and miR-34a were significantly different between AGA and IUGR groups in males, whereas ssc-miR-216, miR-188-5p, miR-144 and miR-10a-5p were significantly different in females. All details of differentially expressed miRNA are shown in electronic supplementary material, table 2. Using RNAhybrid, we found potential target genes of two upregulated and six downregulated miRNAs with their negatively correlated genes, which were also differentially expressed between IUGR and AGA. Finally, 631 mRNA-miRNA pairs were used for further analysis. Our analysis showed that two upregulated miRNAs (miR-885-3p and miR-34a) can potentially target 108 and 145 downregulated genes, respectively. Some of these transcripts belong to important pathways such as HIF-1 signalling pathway (*TF, HIF1A, PIK3R1, IGF1R, PIK3CB*), ferroptosis (*TF*), citrate cycle, glycolysis/gluconeogenesis, PPAR signalling pathway (*ACADM, PCK1, PGM1* and *SUCLA2*), regulation of lipolysis in adipocytes (*ABHD5, PIK3CB* and *PIK3R1*) and TGF-beta signalling pathway (*ROCK1* and *SMAD6*) (figure 5). Six other miRNA which were downregulated in IUGR groups may potentially target 378 transcripts which are enriched in many pathways including citrate cycle (TCA cycle), HIF-1 signalling pathway, ferroptosis, glycolysis/gluconeogenesis, sphingolipid metabolism, ErbB signalling pathway, PPAR signalling pathway, fatty acid elongation and biosynthesis of unsaturated fatty acids (figure 5).

### 2.3. Metabolome analysis

Finally, 742 hepatic metabolites were used for further analysis. Annotation of these metabolites based on KEGG and the Human Metabolome Database (HMDB) identified 599 of 742. The nine most enriched metabolic pathways were arginine biosynthesis, aminoacyl-tRNA biosynthesis, histidine metabolism, arginine and proline metabolism, taurine and hypotaurine metabolism, glycerophospholipid metabolism, sphingolipid metabolism, lysine degradation and linoleic acid metabolism. At a 10% FDR threshold, 83 of 742 metabolites were significantly different in at least one of the group comparisons (see electronic supplementary material, table S3). Most of these metabolites belong to the categories of sphingolipids (33/83), phospholipids (31/83), diglycerid/triglycerids (4/83) and amino acids (3/83).

Forty-one metabolites were differentially altered between the AGA and IUGR groups, including 16 metabolites that were more abundant in the IUGR group, whereas 25 were more abundant in the AGA group. A particular metabolite, omega-linoleoyloxy cer(t18:1(6OH)/26-28:0) with different formulaes, belonging to the sphingolipid category, was frequently found in higher abundance in the IUGR group. Most of the significant metabolites with higher abundance in the IUGR group belong to the nonpolar phase with a positive ionization mode. Only five metabolites belonging to the phosphatidylethanolamine category showed gender differences, including octadecatetraenoate, which was higher in males. In males, 65 altered metabolites were found between AGA and IUGR groups. In the male IUGR group, only 7 metabolites belonging to the sphingolipid class (omega-linoleoyloxy cer(t18:1(6OH)/26-28:0), g-butyrobetaine and 5-hydroxy-DL-tryptophan) were upregulated. Other 58 metabolites were detected at higher levels in male AGA. When AGA and IUGR were compared in female, only 7 metabolites were differentially abundant. Two out of these 7 metabolites were upregulated in IUGR and 2 metabolites (arsenous acid and dihydrothymine) in the AGA group. The details and the number of metabolites that changed between groups and their overlaps are shown in electronic supplementary material, table 3 and figure 2*b*.

### 2.4. Pathway analysis of different metabolites and transcripts

**Figure 6.**
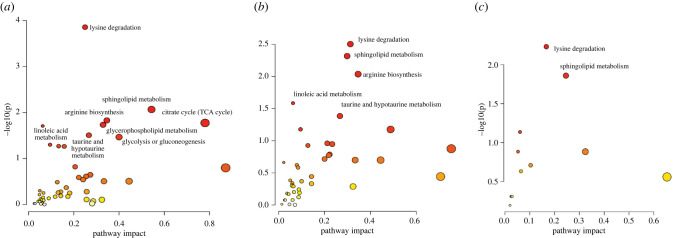
Metabolic pathways analysis of integrated metabolites and transcripts detected in fetal liver (*a*) when comparing AGA and IUGR groups, (*b*) when comparing between male AGA and IUGR fetuses and (*c*) when comparing between female AGA and IUGR fetuses. The significant pathways at *p* < 0.05 were labelled.

To integrate the common pathways from metabolite and transcript data, the lists of significant differentially expressed genes (DEGs) and significant differentially expressed metabolites (DEMs) that were annotable were used for the analysis. Focusing only on metabolic pathways, eight significant pathways (*p* < 0.05) were identified including lysine degradation, sphingolipid metabolism, arginine biosynthesis, citrate cycle (TCA cycle), glycerophospholipid metabolism, linoleic acid metabolism, taurine and hypotaurine metabolism and glycolysis/gluconeogenesis (figure 6*a*). Five metabolic pathways (lysine degradation, sphingolipid metabolism, arginine biosynthesis, linoleic acid metabolism, and taurine and hypotaurine metabolism) were significantly enriched when AGA and IUGR were compared in males (figure 6*b*). Only lysine degradation and sphingolipid metabolism were enriched when AGA and IUGR were compared in females (figure 6*c*).

### 2.5. Integration and identification of biosignatures specifying the IUGR and AGA fetuses

**Figure 7.**
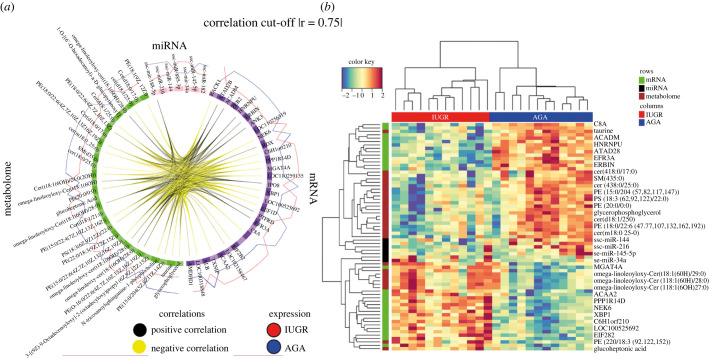
Circos plot and heat map depicting the molecular features identified using DIABLO and their correlation. (*a*) Circos plot demonstrates the biosignature from three datasets, including miRNAs, mRNAs and metabolites associated with to IUGR and AGA. The selected biomarkers from each data type were represented in the outer cycle. The black line indicates a positive correlation, whereas the yellow line represents a negative correlation. The red and blue lines represent the abundance of the features in IUGR and AGA, respectively. (*b*) The heat map of the correlation matrix calculated based on the features selected by DIABLO shows strong groups of highly correlated features. Some of these features are labelled on the left.

After pre-processing and filtering, data from 24 animals (12 from IUGR and 12 from AGA) were considered for further downstream analyses. Finally, we combined transcriptome data (6,284 mRNAs, 40 miRNAs) and metabolome data (742 metabolites) from the liver of IUGR and AGA fetuses. Significant biosignatures were predicted from the dataset using the mixOmics platform. To investigate data variation between IUGR and AGA, we performed a discriminant analysis using sparse partial least square discriminant analysis (SPLS-DA) available in the mixOmics R package. The analysis resulted in the selection of the most discriminating features between IUGR and AGA, including 7 miRNAs, 30 mRNAs and 30 metabolites. Only one component achieved an error rate less than 0.01. A Circos plot shows the correlation between the different omics blocks (figure 7*a*; correlation threshold: |*r*| > 0.75). The heatmap of the biosignature panel containing mRNAs, miRNAs and metabolites shows that the IUGR or AGA group form clusters separately (figure 7*b*). All features selected by DIABLO confirm the major differentially expressed mRNAs, miRNAs and metabolites between fetal groups. Metabolites such as omega-linoleoyloxy cer(t18:1(6OH)/27-29:0 were identified in the panel and correlated strongly positively with the acetyl-CoA Acyltransferase 2 gene (*ACAA2*), whereas they correlated strongly negatively with the acyl-CoA dehydrogenase medium chain gene (*ACADM*). By contrast, *ACAA2* was negatively correlated and *ACADM* was positively correlated with phosphatidylethanolamine (PE), including PE(15:0/22:6(4Z,7Z,10Z,13Z,16Z,19Z)), PE(15:0/20:4(5Z,8Z,11Z,14Z)), PE(18:0/22:6(4Z,7Z,10Z,13Z,16Z,19Z)), PE(15:0/22:4 (7Z,10Z,13Z,16Z)). The activated receptor for activated C kinase 1 (*RACK1*) was the most conspicuous gene, positively correlated with omega-linoleoyloxy cer(t18:1(6OH)/27-29: 0 and negatively correlated with PE(15:0/22:6 (4Z,7Z,10Z,13Z,16Z,19Z)), PE(15:0/20:4 (5Z,8Z,11Z,14Z)) and PE(18:0/22:6 (4Z,7Z,10Z,13Z,16Z,19Z)).

## 3. Discussion

Prenatal development is an important predisposing factor for perinatal development and postnatal growth [26]. Variability in piglet weight depends not only on maternal nutrition but also on other maternal influences such as uterine capacity, parity and health status. Models of IUGR in farm animals are mostly based on maternofetal stress caused by environmental, nutritional or health conditions [27]. Expression profiling of fetal liver of pig differing in utilization and partitioning of energy will point to metabolic pathways, which affect these physiological properties. In addition, the study of the metabolome during prenatal development is a promising way to identify and characterize parameters of piglet maturity at birth [28]. Ultrastructural studies revealed that the fetal pig liver exhibits specific hepatic metabolic competence and hepatopoietic activity at 40–80 dpc, followed by mainly glycogen accumulation [25]. Accordingly, it is of interest to investigate the particular phase of metabolic activity in liver development between IUGR and AGA fetuses, and the focus of this study was on samples derived at 63 dpc. Here, we identified more than 1000 fetal liver transcripts associated with IUGR, including metabolic and non-metabolic pathways.

The HIF-1 signalling pathway and Hippo signalling were among the most dysregulated pathways during embryogenesis or IUGR outcome [3,18,29]. The Hippo signalling pathway has been reported to play an early and essential role in mammalian embryogenesis [30]. HIF-1 is an important transcriptional regulator that mediates cellular responses to hypoxia in mammals [31]. Our present study showed that many transcripts belong to the HIF-1 signalling pathway, including *HIF1A, TF, ALDOB, PGK1, EGLN3, PRKCA* and *EGFR*. Interestingly, we found transcripts regulated in different directions in the IUGR and AGA groups, including a decrease in the level of *HIF1A* in IUGR, whereas the transcript level of *EGLN3* increased. HIFs are regulated posttranslationally by oxygen-dependent hydroxylation of proline residues by prolyl hydroxylase domain protein (PHD), which targets HIF-1*α* for degradation [32]. *EGLN3* is a one of isoforms of PHD and was upregulated in IUGR, possibly to compensate for lower PHD activity caused by oxygen deprivation. Transcriptional upregulation of *EGLN3* has been shown to regulate the HIF response under low oxygen conditions and ensure cell survival under hypoxia [33]. Other transcripts belonging to glycolysis/gluconeogenesis are also associated with HIF-1 signalling pathways (figure 4*a*). Hypoxia can induce or reprogram metabolism to maintain bioenergetic homeostasis during hypoxia [34]. Together with our previous study, HIF-1 signalling and Hippo signalling were shown to play important roles in both skeletal muscle and metabolic process in the liver of IUGR fetuses [3].

Iron-dependent lipid peroxidation (ferroptosis) mediates programmed cell death. Four ferroptosis-related genes, including *ACSL3, ACSL4, GCLM* and *TF*, were decreased in the IUGR group, whereas others, *GPX4, SLC3A2* and *SLC39A14*, were increased, the former promoting ferroptosis and the latter limiting it. The result suggests that ferroptosis in IUGR may be slowed down by an increase in *GPX4, SLC3A2* and *SLC39A14*, leading to a decreased formation of lipid peroxide or iron transport. In fact, glutathione peroxidase 4 (GPX4), which converts lipid peroxides to non-toxic lipid alcohols, is the primary cellular mechanism controlling ferroptosis [35]. Furthermore, solute carrier transporters, including SLC3A2 and SLC39A14, have been shown to limit ferroptosis by reducing iron accumulation. By contrast, ACSL4 and other members of the long-chain acyl-CoA synthetase family, GCLM and TF limit ferroptosis by reducing or blocking GPX4 or impeding iron transport [36,37].

Furthermore, when comparing IUGR and AGA, ribosome and ferroptosis were more enriched in males than females. This may suggest that male IUGRs attempt to prioritize growth through upregulated ribosomal transcripts while upregulating apoptosis or ferroptosis, as reported in a previous review on sex-specific adaptations to a change in the *in utero* fetal environment [38].

Another pathway of interest was Cushing's syndrome. We found genes such as *AGTR1, CDKN2B, CRHR1, EGFR, FH* and *LDLR* were upregulated in IUGR, while *RB1, USP8, APC, GSK3B* and *ITPR1* were downregulated in IUGR fetuses. Cushing's syndrome is a disorder that occurs when too much of the hormone cortisol is produced over a long period of time. During pregnancy, maternal cortisol levels increase and have a positive effect on neural development [39]. However, overexposure to glucocorticoids resulted in lower birth weight, an unfavourable metabolic profile and a behavioural phenotype in adulthood in the offspring [40].

Many biological processes and KEGG pathways involved with energy metabolism (citrate cycle, glycolysis/gluconeogenesis) and lipid metabolism (lipid oxidation, fatty acid oxidation, lipid transport and fatty acid degradation) were significantly changed when comparing IUGR and AGA fetuses. Gluconeogenesis is a pathway of glucose synthesis from non-carbohydrate precursors during fasting, starvation or IUGR conditions to maintain blood glucose levels. Studies of IUGR in sheep showed increased expression of gluconeogenic genes, including the rate-limiting phosphoenolpyruvate carboxykinases 1 and 2 (*PCK1* and *PCK2*) [27]. We found that transcripts such as *GAPDH, ALDOB, ENO1* and *PGK1*, which are part of glycolysis/gluconeogenesis, were more highly expressed in the fetal liver with IUGR. Glycolysis, a metabolic pathway that breaks down glucose, is critically regulated by insulin secretion [41]. Fetuses with IUGR have low insulin concentrations [42], which partially explains the low glycolysis along with the upregulation of gluconeogenesis in IUGR. Other metabolic pathways associated with IUGR are the citrate cycle (TCA cycle) including *ACO1, FH, IDH1* and *MDH1*, which are overexpressed in the IUGR liver. A previous study reported that skeletal muscle in IUGR adapts to hypoxaemia and hypoglycaemia by decreasing the activity of complex I and TCA cycle enzyme [43]. By contrast, fetuses with IUGR undergo early activation of hepatic glucose production, an ATP- and substrate-intensive process that is activated to counteract hypoglycaemia [11]. These observations suggest that various metabolic adaptations in IUGR are tissue-specific [6]. Lipids in the liver are associated with a number of biological functions, including the provision of energy, as a major structural component of membranes, mostly belonging to the glycerophospholipids, and as important signalling lipids such as the sphingolipids. Maternal lipid metabolism is involved in biological processes for cell growth and development, cell signalling and the development of critical structural and functional features of the feto-placental unit [44]. Fatty acids are an essential energy source for the fetus, while phospholipids are important as cell membrane components and for tissue development [45]. Fatty acid binding protein (FABP1) is crucial for fatty acid uptake and intracellular transport and also plays an important role in regulating lipid metabolism and cellular signalling pathways, and as a cellular antioxidant [46]. The expression of *FABP1*, which belongs to the PPAR pathway, was significantly lower in the IUGR groups compared to AGA (*p* < 0.003) and was particularly low in the male IUGR group. For other signalling pathways such as lipid degradation, transcripts including *FABP1, ACADL, ACADM, ACADSB, GCDH, ACSL4* and *ACSL3* were also less expressed in the IUGR groups.

Differentially abundant transcripts and metabolites were found that were mutually confirmed to belong to the same metabolic pathways. Analyses of the metabolic pathways of differentially expressed transcripts and differentially abundant metabolites showed, in particular, shifts in the lipid metabolism in IUGR and AGA (figure 6*a*). Interestingly, when IUGR and AGA were compared, a greater difference in metabolic characteristics was observed in males than in females. These sex bias phenomena can be observed not only from the lower weight of male IUGR compared with female IUGR (figure 1), but also in a number of transcript and metabolite changes (figure 2*a,b*). Accordingly, sex-specific developmental dynamics were observed in a previous study focusing on the liver transcriptome of IUGR piglets [47]. In particular, male IUGR piglets are more susceptible to impaired metabolic homeostasis [47]. Together with our data, it indicates that the sexual dimorphism associated with IUGR occurs early in the embryonic period and that IUGR is more pronounced in males, especially in the metabolic system. Elements of the sphingolipid pathway are not only components of the cell membrane, but also bioactive lipids that are signalling molecules in cellular processes such as differentiation and apoptosis [48,49]. Sphingolipid metabolism is required for the maintenance of normal pregnancy [50,51]. As shown in this study, besides phospholipids, sphingolipids were the major group of lipids that differed between IUGR and AGA. Ceramides are intermediates of sphingolipid metabolism obtained by both *de novo* synthesis and via recovery pathways. We found that some of these intermediates (Cer(d18:)/Cer(t18:)/Cer(m18:)/Cer(t15:) are lower in IUGR than in AGA fetuses. Other metabolites that also belong to the sphingolipids of the main class of ceramides are omega-linoleoyloxy-Cer(t18:1(6OH)/26-19:0), but these are more abundant in IUGR than in AGA. Interestingly, these differences between IUGR and AGA are more pronounced in male than in female fetuses. In this study, we found significantly lower taurine levels in IUGR compared to AGA (*p* < 0.0005) and especially lower levels in male IUGR. Taurine, a sulfur-containing organic acid with various cellular and physiological functions [52], is synthesized in the liver from methionine/cysteine in adults, while fetuses depend on taurine supplied by mothers via the placenta [53]. This indicates that insufficient maternal nutrient supply to fetus is the main cause of impaired development.

MiR-34a, one of the two miRNAs shown to be increased in the IUGR groups in this study, has been found to be abundant in the placenta, maternal circulation or muscle tissue, associated with the pathophysiology of IUGR or preeclampsia [18,20]. The same study also reported that miR-34a is induced by hypoxia in choriocarcinoma cells [20]. The miR-34a is also involved in fat and glycogen metabolism under hypoxia stress [54]. Our previous study using muscle tissues of the same animals revealed upregulation of miRNA, including miR-34a and miR-210, in the IUGR group [3]. By contrast, miR-210, which has been mostly reported to be involved in hypoxia [19,55], was not differentially expressed in liver, as shown here. This tissue-specific transcriptional responses of mR-34a and miR-210 suggest differential sensitivity and function in the context of IUGR. In this study, miR-34a predicted targets belong to transcripts enriched in HIF-1 signalling pathway (*TF, PIK3R1, IGF1R* and *PIK3CB*) and metabolic pathways (*PCK1, PGM1, SUCLA2, ROCK1, SMAD6, ACADM* and *ABHD5*) (figure 5). MiR-885-3p is another miRNA that was also upregulated in the IUGR groups in this study. The predicted target transcripts of miR-885-3p were enriched in the HIF-1 signalling pathway (*TF* and *HIF1A*), TGF-beta signalling pathway (*TGFB1* and *SMAD6*) and citrate cycle (*PCK1* and *SUCLA2*). Six miRNAs (miR-216, miR-188-5p, miR-144, miR-10a-5p, let-7 g-5p and miR-182) were downregulated in the IUGR groups. Many placental miRNAs have been linked to IUGR, including let-7 g, which plays a role in fetal growth and development [56]. Our previous study showed that hepatic expression of let-7 family members negatively correlates with blood glucose and triglyceride levels [23]. A recent study showed that overexpression of let-7 reduces glucose production in primary hepatocytes of obese individuals [57]. Moreover, hepatic administration of let-7 improves hyperglycaemia and glucose homeostasis in diabetic mice [57]. In our study, glucose levels tended to be higher in the IUGR liver, while significantly higher triglycerides were observed in the IUGR liver, as well as downregulation of let-7 g-5p and an increase in key gluconeogenic genes.

Fetal and placental growth in pigs is influenced by many factors including genetic, epigenetic and environmental factors, even by the sex status of adjacent fetuses or the intrauterine position [58]. It has been reported that fetuses positioned toward the uterine-tubal junction are larger than fetuses positioned toward the cervix [59]. In addition, fetal weight has been reported to vary within a litter due to limited uterine capacity [60]. Overall, the causal reason for IUGR, even in our study with IUGR and AGA fetuses selected from the same mother (same-sex divergent full siblings) i.e. the same environment, apart from intrauterine position, remains to be elucidated. Measurements of umbilical blood concentrations and uterine and umbilical blood flow rates provide further information on whether the metabolic changes are due to nutrient deficiency, hypoxia or both. We have compiled a comprehensive systems biology study of omics data that reveals interactions and changes in the metabolic system associated with IUGR. Our multi-omics correlation and network analysis identified a bio-signature that differs between IUGR and AGA. In particular, the dependency between activated genes and metabolites plays an important role in metabolic homeostasis and shows distinct aberrations in IUGR. Most of the changes during mid-pregnancy in the IUGR liver involved lipid metabolism, especially in the category of sphingolipids and phospholipids, at both the transcript and metabolite levels. The HIF-1 pathway, the Hippo pathway and the functional pathways of ribosomes, glycolysis/gluconeogenesis and ferroptosis, as well as Cushing's syndrome were significantly altered when comparing IUGR and AGA. In addition, significant miRNAs with target transcripts enriched in the above pathways were identified, particularly miR-34a. Sex-specific phenomena were observed in both transcripts and metabolites and occur early in the embryonic stage, being more pronounced in males, especially in the metabolic system. This suggests that sex-specific adaptations in the liver occur in the metabolic system at mid-pregnancy (63 dpc).

## 4. Material and methods

### 4.1. Animals and sample collection

In this study, liver samples were used from the same animals previously used for muscle tissue analyses [3]. As previously described, one sire and 11 dams from a cross of German Landrace and Pietrain were used and a total of 118 fetuses were obtained at day 63 post-conception [3]. Discordant sibling pairs representing fetal weight extremes were selected from the 118 F2 fetuses. Fetuses that had less than two s.d. of the mean weight of the littermates were classified as intrauterine growth restricted (IUGR), while sex-matched littermates with weight close to the mean were classified as appropriate for gestational age (AGA). Based on these criteria, a total of 12 sibling pairs from 8 dams were selected for this study (IUGR; *n* = 12 including 7 males and 5 females; AGA; *n* = 12 including 7 males and 5 females).

In addition, we calculated relative fetal weight (%) as [(fetal weight−mean litter fetal weight)/mean litter fetal weight] × 100 according to a formula previously used to characterize IUGR using birth weight to assess mortality risk [61–63]. During the experiment, pigs had ad libitum access to feed (Trede and von Pein, Itzehoe, Germany) and water in standard housing of the FBN experimental station. After opening of the uterus, the fetuses were sequentially retrieved. The umbilical cord was cut about 2 cm from the umbilicus of each fetus, and the fetus was exsanguinated. Sex was determined by visual inspection of the external genitalia (clearly visible at this age) and recorded. Subsequently, the fetus was weighed on a Sartorius LC621P scale. Liver tissue from AGA and IUGR fetuses was immediately frozen in liquid nitrogen and stored at −80°C until RNA or metabolome extraction.

### 4.2. RNA isolation and gene expression profiling

Total RNA was isolated from ground livers using Tri-Reagent and processed using RNeasy Mini-Kits (Qiagen) and on-column DNase treatment. Electrophoresis in 1% agarose gels and spectrophotometric measurements using a Nano Drop ND-1000 spectrophotometer (PEQLAB) were performed to determine RNA integrity and quantification. Finally, additional measurements were performed using the Agilent 2100 Bioanalyzer (Agilent) and Agilent kits for RNA quantification.

Affymetrix microarrays (Affymetrix Snowball, Geo Platform GPL16569) with 47 880 probe-sets were used. 500 ng RNA of each of the 24 samples was transcribed first into cDNA and then into biotin-labelled cRNA and hybridized onto the arrays using the Affymetrix WT plus Expression Kit and the Genechip WT terminal labelling and hybridization kit according to the manufacturer's instructions (Affymetrix, Santa Clara, CA, USA). Hybridization, washing and scanning of the arrays were performed on Affymetrix hybridization ovens, fluidics stations and scanners and the Affymetrix GCOS1.1.1 software was used for quality control. The Expression Console software was used to obtain expression values by using robust multichip average (RMA) normalization and detection above background (DABG) algorithms. Expression values were further filtered to exclude transcripts with low signals and probe sets that were present in less than 75% of the samples. 10 086 probe sets passed the quality filtering and were used for further analyses. The expression data are available in the Gene Expression Omnibus public repository with the GEO accession number (GSE202677: GSM6128313- GSM6128336).

### 4.3. miRNA and validation of mRNA by qPCR

Selected differentially expressed mRNA transcripts and miRNAs were quantified in the liver from AGA and IUGR groups by qPCR using the Fluidigm BioMark HD System. The cDNA synthesis of miRNA and mRNA was performed according to a previous study [64]. Briefly, 100 ng of total RNA were poly(A) tailed and reverse transcribed using 1 unit of poly(A) polymerase 1 µM (BioLab), RT-primers (CAGGTCCAGTTTTTTTTTTTTTTTVN where V is A, C and G and N is A, C, G and T), 0.1 mM of NTPs, 100 units of MuLV reverse transcriptase (Invitrogen). The reaction was incubated at 42 °C for 1 h followed by 95 °C to inactivate the enzyme. In total 40 miRNA from 24 samples with two replicates each were used for qPCR with the Fluidigm BioMark HD System. Specific target amplification (STA) was done per manufacturer's recommendations. Pre-amplification sample mixtures were prepared using PreAmp Master Mix (Fluidigm PN 1005581) containing 1.25 µl of cDNA, 1 µl PreAmp Master Mix and 0.5 µl Pooled Delta Gene Assay Mix (500 nM) containing DNA-suspensions buffer and primers mixes (electronic supplementary material, file S1) in 5 µl total volume. The preamplification reaction was incubated at 95°C for 2 min, followed by 10 cycles at 95°C for 15 s and 60°C for 4 min. The preamplification reaction was cleaned up using exonuclease I, followed by 10 × dilution of STA with DNA suspension buffer (TEKnova, PN T0221). Fluidigm quantitative measurement runs were carried out with 48.48 dynamic arrays (Fluidigm Corporation, CA, USA) according to manufacturers instructions. In brief, 2.5 µl of 2 × SsoFast Evagreen Supermix with Low ROX, 0.25 µl 20 × sample-loading reagent and 2.25 µl of treated samples were prepared. Separately, an assay mixture was prepared for each primer pair and this included 2.25 µl of DNA Suspension buffer, 0.25 µl of 100 µM forward and reverse primer and 2.5 µl of 2 × assay-loading reagent. The dynamic arrays were first primed with control line fluid and then loaded with the sample and assay mixtures via the appropriate inlets using an IFC controller. The array chips were placed in the BioMark Instrument for PCR at 95°C for 10 min, followed by 30 cycles at 95°C for 15 s and 60°C for 1 min. The data were analysed with real-time PCR analysis software in the BioMark HD instrument (Fluidigm Corporation, San Francisco, CA). The internal controls of cel-miR-39-3p, 5S and 18S were used for miRNA and Actin beta (*ACTB*), *YMHAZ* and *RPS11* for mRNA. All these endogenous reference genes were unaffected by factors used in the study. Data analysis was done by 2^−Δ*C*t^ method. The primer sequences are listed in electronic supplementary material, table S4.

### 4.4. Metabolomic analysis

Liver samples are analysed according to protocols established at the FBN Core Facility Metabolomics [65]. Briefly, liver tissue samples were collected and frozen in liquid nitrogen and stored at −80°C until extraction. The samples were ground and homogenized before being split for extraction. The polar and non-polar layers were separated and dried under nitrogen flow at room temperature and stored until analysis. After reconstitution, the nonpolar phase and the polar phase were analysed in positive and negative ionization mode by RP ultra-high performance liquid chromatography-tandem mass spectrometry (UHPLC-MS/MS) (Vanquish UHPLC-System with heated electrospray ionization (HESI) QExactive plus Orbitrap mass spectrometer; Thermo Scientific, Waltham, USA). Identification and relative quantification of individual lipid species were performed at the production level (MS/MS fragmentation) using LipidSearch Software (Thermo Scientific, Waltham, MA, USA), and annotation of small metabolites was performed using Compound Discoverer 3.2 Software (Thermo Scientific, Waltham, MA, USA). In total 990 metabolites, from both polar and non-polar part, were identified. The metabolome was further filtered, normalized by logarithmic transformation, centred on the mean and divided by the square root of the standard deviation of each variable (Pareto scaling) using MetaboAnalyst 4.0 [66]. Finally, 742 metabolites were used for further analysis. The Human Metabolome Database (HMDB; http://www.hmdb.ca) and KEGG Database were used to identify metabolites by matching the molecular weight, numeric mass (*m*/*z*) values, retention times and ion mode.

### 4.5. Differentially expressed mRNA, miRNA and metabolites

To determine whether there were differences in liver gene expression and liver metabolites based on fetal weight groups, the normalized expression and metabolite data served as dependent variables for variance analysis using JMP Genomics 9.0 (SAS Institute, Cary, NC, USA). The mixed model analysis procedure under JMP Genomics 9.0 (SAS Institute, Cary, NC, USA) was used for statistical analysis. A linear model was applied that included fetal weight group (IUGR and AGA) and sex (male and female) as fixed effects and mother as a random effect. The Tukey-Kramer post hoc test (type III) was calculated to adjust each comparison for all effects, IUGR versus AGA or IUGR versus AGA for female or IUGR versus AGA for male. We considered FDR less than 0.1 as significant threshold for mRNA and metabolite. Due to a small number of miRNA input, we considered significance thresholds of miRNA at *p* < 0.05.

### 4.6. Prediction of miRNA target genes and their correlation analysis

To investigate the downstream target mRNAs for differentially expressed miRNAs (eight miRNA) between IUGR and AGA fetuses, 17 065 3′-UTR sequences, 16 857 5′-UTR sequences and 20 310 coding sequences were extracted from the Sus scrofa genome sequence (Sscrofa11.1) based on Ensembl annotation v. 102. Using the sequences of mature miRNAs, RNAhybrid v. 2.1.2 was used to predict the target genes of differentially expressed miRNAs. The parameters were set for a single hit per target, a human-based assumed *p*-value distribution, a minimum free energy (MFE) threshold of less than −25 kcal mol^−1^, and a helix restriction of base 2 to 7 [67,68]. Pearson correlation between miRNAs and mRNAs was calculated. Only the predicted target mRNA that were negatively correlated with miRNA and that were also differentially expressed between IUGR and AGA fetuses were used for further analyses.

### 4.7. Data integration of the metabolome, mRNA and miRNA

The normalized metabolites, mRNAs and miRNAs were used as input for further analysis. In order to identify a highly correlated multi-omics signature discriminating between IUGR and AGA groups, the multi-block discriminant analysis with DIABLO (Data Integration Analysis for Biomarker discovery using a Latent cOmponents) embedded in the R package ‘mixOmics’ (v. 6.6.2) was used [69,70]. Transcripts and metabolome data were used as input for identifying the molecular drivers for IUGR traits.

To assess the number of parameters, the global performance, the balanced error rate (BER), to select the optimal metric distance, and to define the number of components kept for our block.splsda analysis, we computed the evaluation criteria using the perf() function from DIABLO. As input arguments we used our block.splsda object (without variable selection), Mfold validation (*n* = 10), repeated cross-validation (50 repetitions). We fine-tuned our model using tune.block.splsda() function, and determined the optimal number of variables kept for our final block.splsda analysis. The output variable $choice.ncomp integrates the centroids.dist distance as well as the BER and indicates the optimal number of components for the final DIABLO model.

## Data Availability

The expression data are available in the Gene Expression Omnibus public repository with the GEO accession number (GSE202677: GSM6128313–GSM6128336). The data are provided in electronic supplementary material [71].
